# Natural vertical transmission of dengue viruses by *Aedes aegypti* in Bolivia

**DOI:** 10.1051/parasite/2011183277

**Published:** 2011-08-15

**Authors:** G. Le Goff, J. Revollo, M. Guerra, M. Cruz, Z. Barja Simon, Y. Roca, J. Vargas Florès, J.P. Hervé

**Affiliations:** 1 Institut de Recherche pour le Développement (IRD), UR 016 La Paz Bolivie; 2 Centro Nacional de Enfermedades Tropicales (CENETROP) Santa Cruz de la Sierra Bolivia; 3 Universidad Autónoma Gabriel Rene Moreno Santa Cruz de la Sierra Bolivia

**Keywords:** *Aedes aegypti*, vertical transmission, dengue virus, PCR, *Aedes aegypti*, transmission verticale, dengue, virus, PCR

## Abstract

The natural transmission of dengue virus from an infected female mosquito to its progeny, namely the vertical transmission, was researched in wild caught *Aedes aegypti* during an important outbreak in the town of Santa Cruz de la Sierra, Bolivia. Mosquitoes were collected at the preimaginal stages (eggs, larvae and pupae) then reared up to adult stage for viral detection using molecular methods. Dengue virus serotypes 1 and 3 were found to be co-circulating with significant higher prevalence in male than in female mosquitoes. Of the 97 pools of *Ae. aegypti* (n = 635 male and 748 female specimens) screened, 14 pools, collected in February-May in 2007, were found positive for dengue virus infection: five DEN-1 and nine DEN-3. The average true infection rate (TIR) and minimum infection rate (MIR) were respectively 1.08% and 1.01%. These observations suggest that vertical transmission of dengue virus may be detected in vectors at the peak of an outbreak as well as several months before an epidemic occurs in human population.

## Abstract (fr)

La transmission naturelle du virus de la dengue d’un moustique femelle infecté à sa descendance, à savoir la transmission verticale, a été recherchée chez des *Aedes aegypti* sauvages capturés lors d’une importante épidémie dans la ville de Santa Cruz de la Sierra (Bolivie). Les moustiques collectés aux stades préimaginaux (oeufs, larves et nymphes) ont été mis en élevage jusqu’au stade adulte pour la détection de particules virales de la dengue en utilisant une méthode de biologie moléculaire. La détection moléculaire a révélé la co-circulation de deux sérotypes de la dengue, DEN-1 et DEN-3, avec une prévalence significativement plus élevée chez les moustiques mâles que chez les moustiques femelles. Parmi les 97 pools d’*Ae. aegypti* testés (n = 635 mâles et 748 femelles), 14 pools, récoltés entre février et mai 2007, ont été trouvés positifs pour un sérotype de la dengue : cinq en DEN-1 et neuf en DEN-3. Le taux d’infection moyen (TIR) et le taux minimum d’infection (MIR) étaient respectivement de 1,08 % et 1,01 %. Ces observations suggèrent que la transmission verticale des virus de la dengue peut être détectée chez les vecteurs aussi bien au pic de l’épidémie dans la population humaine que plusieurs mois avant le début de l’épidémie.

Dengue is currently one of the most important human arbovirus infections worldwide. The only prevention tool for dengue is vector control as there is no vaccine, nor any efficient drugs available. Dengue, which is transmitted by *Aedes aegypti*, has been in constant expansion since the end of the 1980s in Bolivia. *Ae. aegypti* has re-established itself in the city of Santa Cruz de la Sierra recently and the presence of dengue in Bolivia has been documented from 1987. Four very well documented epidemics took place since 1999, where at least one serotype was isolated every year (Roca *et al.*, 2009). Although vertical transmission of the dengue virus from mosquito females to their progeny has been suggested since the 1970s, it was only demonstrated in nature recently (Hull *et al.*, 1984). The first evidence came from the laboratory (Rosen *et al.*, 1983). In natural conditions, attempts to isolate the virus from immature stages in South America were either negative (*e.g.* in Colombia, Romero-Vivas *et al.*, 1998) or positive (*e.g.* in Guyana, Fouque & Carinci, 1996). In this study we investigated the occurrence and prevalence of vertical transmission and maintenance of the virus in the city of Santa Cruz, Bolivia.

## Materials and methods

In January 2007, higher number of consultations was recorded in the city of Santa Cruz and numerous cases of dengue were immunologically confirmed. Entomological study was performed in February 2007 in houses having indicated cases of dengue within the last ten days. 11 houses distributed mainly in the city centre were prospected for larvae and pupae and followed-up using ovitraps. A survey was performed in May 2007 with 100 groups of five to 12 houses randomly selected in the whole city of Santa Cruz. Oviposition traps consisted of black plastic cans partially filled with water. Two hardboard paddles were placed in each ovipot for oviposition. In each house, four ovitraps were placed in sheltered areas (two inside and two outside the buildings) and examined weekly. Larvae and pupae were collected from the water of all containers by passing out the water through a sieve. Hatching larvae from egg paddles and field collected larvae of *Ae. aegypti* were reared separately. Adult mosquitoes (one to seven days after emergence) were separated by sex, pooled in vials and stored at - 70 °C. Pools ranging from three to 15 mosquitoes were ground in sterile phosphate buffered saline (PBS) (mean ± standard deviation of individuals = 14.3 ± 2.0). Total RNA extracted from mosquitoes was converted in cDNA (RT) and following by a semi-nested PCR (Harris *et al.*, 1998). Sterile water was used as a negative PCR control. In any case, the negative control samples that were coamplified in each PCR procedure did not yield any product. The positivity of a pool of mosquitoes was confirmed by amplifying a product PCR. The amplification products were identified by their molecular weights: 482, 119, 290 and 389 bp for dengue serotype 1, 2, 3 and 4, respectively.

The minimum infection rate (MIR) was defined as the ratio of the number of positive pools to the total number of mosquitoes. MIR assumes that only one infected individual is present in a positive pool. Consequently, we also used the true infection rate (TIR) defined as the estimated percentage of infected mosquitoes. Because TIR was calculated from pools of different sizes, we applied the maximum likehihood procedure developed by Katholi *et al*. (1995) using available algorithms that consider variations in pool size (Gu *et al.*, 2004).

## Results

A total of 46 pools of males and 51 pools of females of *Ae. aegypti*, representing respectively 635 males and 748 females of mosquito, were tested by PCR to reveal the presence of the serotypes of dengue RNA. Fourteen pools of mosquitoes, 11 pools of males and three of females revealed the presence of at least one dengue virus, serotype 1 or serotype 3 (Table I). No double infection was observed. The TIR and the MIR were estimated at 1.08 % (95 % confidence interval: 0.56-1.86 %) and 1.01 %, respectively. The specific TIR were estimated at 0.37 % (0.11-0.87 %) for dengue 1 and 0.68 % (0.29- 1.31 %) for dengue 3. In February 2007, the serotype 3 alone was observed among four pools of males. One from a pool of mosquitoes emerged from 20 positive breeding sites collected in seven positive houses and three among eggs collected by ovitraps. The survey in May 2007 collected larvae and/or pupae of *Ae. aegypti* in 1,057 positive breeding sites distributed in 421 houses. Two serotypes of dengue were observed in equal parts, as well as in pools of males or females: five pools of DEN-1 and five pools of DEN-3. Interestingly, some differences were observed according to the sex of the studied individuals. Globally, the MIR for males and females were 1.73 % and 0.40 % (p < 0.05), respectively. Similarly, in May, the MIRs were estimated at 4.02 % and 1.05 % (p < 0.05), respectively, for males and females. On the other hand, in the city centre in February 2007, the difference between males and females (0.87 % and 0.00 %, respectively) was not significant (p = 0.07).

**Table I.** Numbers of mosquitoes, pools tested and dengue isolates in 2007 in the city of Santa Cruz de la Sierra, Bolivia.

Date collection	Method of capture	Sex	No. Mosquitoes tested	No. pools tested	No. positive pools of DEN-1	No. positive pools of DEN-3
February	Larval and pupal	M	106	8	0	1
	survey	F	123	9	0	0
	Ovitraps	M	355	24	0	3
		F	340	23	0	0
May	Larval and pupal	M	174	14	3	4
	survey	F	285	19	2	1

Total			1,383	97	5	9

## Discussion and conclusion

The efficiency of vertical transmission of serotypes of dengue was demonstrated in populations of vectors in Santa Cruz de la Sierra. This general term indicates that the infection is transmitted to the progeny of mosquitoes, and demonstrates that mosquitoes play not only the role of vectors but also a reservoir of virus (Fontenille *et al.*, 1997; Ibáñez-Bernal *et al.*, 1997). Our understanding of the epidemiology of arboviruses is not complete and in particular as to how the dengue virus persists in unfavourable conditions. Numerous hypotheses have been proposed, but the only one demonstrated is the existence of vertical virus transmission in their vector mosquitoes. Vertical transmission in nature has been estimated occasionally, but this mode of transmission could be more important than usually considered. For detecting vertical transmission, the previous studies using immunofluorescence method detected lower infection rate than in our study (Khin & Than, 1983; Hull *et al.*, 1984; Arunachalam *et al.*, 2008). In Santa Cruz we observed about 1 % of infected vectors in epidemic period. This result was similar to other recent studies in Asia and in America using RT-PCR molecular method (Kow *et al.*, 2001; Ratanasetyuth, 2004; Günther *et al.*, 2007; Cecílio *et al.*, 2009). The molecular detection of the viruses in vectors could be a useful additional tool to detect the presence of a new serotype, possibly at the origin of a new epidemic (Chow *et al.*, 1998). More surprising is the detection of the serotype DEN-1 as early as May 2007 among vectors while this serotype was revealed in patients from Santa Cruz in April 2008, *i.e.* 11 months later (Roca *et al.*, 2009). Even if the period of time between the detection of dengue viral RNA in mosquito and the first human cases is not well quantified, we know it generally occurs within a few weeks (Lee & Rohani, 2005). In the case of our study the first human cases of the disease were declared and/or identified only in the next season of transmission, almost one year after its presence was established in the vector. This example demonstrates that vertical transmission of the dengue is underestimated in epidemics, as well as modelling developed to describe the appearance of dengue among virgin populations.
